# Rapid isolation and immune profiling of SARS-CoV-2 specific memory B cell in convalescent COVID-19 patients via LIBRA-seq

**DOI:** 10.1038/s41392-021-00610-7

**Published:** 2021-05-17

**Authors:** Bing He, Shuning Liu, Yuanyuan Wang, Mengxin Xu, Wei Cai, Jia Liu, Wendi Bai, Shupei Ye, Yong Ma, Hengrui Hu, Huicui Meng, Tao Sun, Yanling Li, Huanle Luo, Mang Shi, Xiangjun Du, Wenjing Zhao, Shoudeng Chen, Jingyi Yang, Haipeng Zhu, Yusheng Jie, Yuedong Yang, Deyin Guo, Qiao Wang, Yuwen Liu, Huimin Yan, Manli Wang, Yao-Qing Chen

**Affiliations:** 1 School of Public Health (Shenzhen), Sun Yat-sen University, Shenzhen, China; 2 Wuhan Institute of Virology, Center for Biosafety Mega-Science, Chinese Academy of Sciences, Wuhan, China; 3 Pulmonary and critical care medicine, The Third People’s Hospital of Dongguan City, Dongguan, Guangdong Province China; 4 Hangzhou ImmuQuad Biotechnologies, Hangzhou, China; 5 Zhejiang-California International NanoSystems Institute, Zhejiang University, Hangzhou, China; 6 Infection and Immunity Center, School of Medicine, Sun Yat-sen University, Shenzhen, China; 7 Guangdong Provincial Key Laboratory of Colorectal and Pelvic Floor Diseases, The Sixth Affiliated Hospital, School of Medicine, Sun Yat-sen University, Guangzhou, China; 8 Guangdong Provincial Key Laboratory of Biomedical Imaging, The Fifth Affiliated Hospital, Sun Yat-sen University, Zhuhai, China; 9 Department of Infectious Diseases, The Ninth People’s Hospital of Dongguan City, Dongguan, Guangdong Province China; 10 Department of Infectious Diseases, The Third Affiliated Hospital of Sun Yat-sen University, Guangzhou, Guangdong Province China; 11 School of Data and Computer Science, Sun Yat-sen University, Guangzhou, China; 12 Key Laboratory of Medical Molecular Virology (MOE/NHC/CAMS), School of Basic Medical Sciences, Shanghai Medical College, Fudan University, Shanghai, China; 13 Shenzhen Branch, Guangdong Laboratory for Lingnan Modern Agriculture, Shenzhen, China; 14 Genome Analysis Laboratory of the Ministry of Agriculture, Shenzhen, China; 15 Agricultural Genomics Institute at Shenzhen, Chinese Academy of Agricultural Sciences, Shenzhen, China; 16 Key Laboratory of Tropical Disease Control (Sun Yat-sen University), Ministry of Education, Guangzhou, China

**Keywords:** Adaptive immunity, Infectious diseases, Infectious diseases

## Abstract

B cell response plays a critical role against SARS-CoV-2 infection. However, little is known about the diversity and frequency of the paired SARS-CoV-2 antigen-specific BCR repertoire after SARS-CoV-2 infection. Here, we performed single-cell RNA sequencing and VDJ sequencing using the memory and plasma B cells isolated from five convalescent COVID-19 patients, and analyzed the spectrum and transcriptional heterogeneity of antibody immune responses. Via linking BCR to antigen specificity through sequencing (LIBRA-seq), we identified a distinct activated memory B cell subgroup (*CD11c*^*high*^ *CD95*^*high*^) had a higher proportion of SARS-CoV-2 antigen-labeled cells compared with memory B cells. Our results revealed the diversity of paired BCR repertoire and the non-stochastic pairing of SARS-CoV-2 antigen-specific immunoglobulin heavy and light chains after SARS-CoV-2 infection. The public antibody clonotypes were shared by distinct convalescent individuals. Moreover, several antibodies isolated by LIBRA-seq showed high binding affinity against SARS-CoV-2 receptor-binding domain (RBD) or nucleoprotein (NP) via ELISA assay. Two RBD-reactive antibodies C14646P3S and C2767P3S isolated by LIBRA-seq exhibited high neutralizing activities against both pseudotyped and authentic SARS-CoV-2 viruses in vitro. Our study provides fundamental insights into B cell response following SARS-CoV-2 infection at the single-cell level.

## Introduction

The emerging COVID-19 pandemic, caused by severe acute respiratory syndrome coronavirus 2 (SARS-CoV-2), has caused enormous global public health damage and economic crises.^1,2^ Speedy development of vaccines and monoclonal antibodies are required to prevent and control SARS-CoV-2 infection.^3–8^ Currently, no therapeutic antibody has been approved for COVID-19 disease.^9^ Humoral immune response plays a critical defensive role in preventing SARS-CoV-2 infection. Neutralizing monoclonal antibodies (mAbs) isolated from convalescent patients’ memory B cells represent a promising intervention for COVID-19 disease. An increasing number of mAbs have been isolated from convalescent COVID-19 patients by single B cell antibody cloning.^10–16^ To better understand the human B cell immune response and antibody response against SARS-CoV-2 would facilitate the development of potent neutralizing mAbs and promote the design of efficient SARS-CoV-2 vaccines.^17^

Antibody diversity and specificity depend on the somatic hypermutation (SHM) and affinity maturation of B cell receptor (BCR) after virus infection.^18,19^ Understanding BCR molecular diversity and evolution following SARS-CoV-2 infection is the key to elucidating the dynamics of the adaptive immune system within individuals. Due to the variable (V), diversity (D) and joining (J) (V (D) J) recombination and SHM, B cells have diverse BCR repertoires.^11,20,21^ Single cell RNA sequencing (scRNA-seq) and single cell VDJ sequencing (scVDJ-seq) provide rapid and efficient methods for acquiring naturally paired BCR heavy and light chain repertoires.^22–24^ To detect BCR-antigen binding interactions, linking BCR to antigen specificity through sequencing (LIBRA-seq) has been developed by utilizing oligonucleotides conjugated to recombinant antigens.^23^ The antibody responses against distinct viruses or pathogens generally function similarly across individuals.^24^

Some BCR antibody clonotypes share the same IGHV and IGLV genes among multiple individuals. These so called, public antibody clonotypes have been identified in HIV,^24^ influenza^25^ and other infections.^26^ As for SARS-CoV-2 infection, researchers have analyzed 294 antibodies and found that *IGHV3-53* was the most frequently used IGHV gene targeting the receptor-binding domain (RBD) of the spike protein,^4^ with same characteristics of *IGHV3-53*: short CDRH3 loops, minimal affinity maturation and high potency, makes it a promising candidate for vaccine design. Knowledge of BCR repertoires diversity can provide insights into how the immune repertoire quickly responds to novel microbial pathogens, and therefore facilitates rational vaccine design. However, the diversity of the BCR repertoires and the B cell transcriptome profiling following SARS-CoV-2 infection at the single-cell level are still largely unknown.

Here, we analyzed the diversity and frequency of the naturally paired immunoglobulin heavy and light chains in five convalescent COVID-19 patients by scRNA-seq and scVDJ-seq, and characterized the non-stochastic BCR clonotypes and public antibody clonotypes. Interestingly, we found that, compared with memory B cells, the activated memory B cell subgroup had a statistically higher percentage of SASRS-CoV-2-specific B cells. Moreover, two antibodies C14646P3S and C2767P3S, isolated via high-throughput LIBRA-seq bound SARS-CoV-2 RBD and exhibited high neutralizing activities against both pseudotyped and authentic SARS-CoV-2 viruses in vitro. Our study is believed shed light on the development of effective vaccination and therapeutic antibodies against COVID-19 pandemic.

## Results

### Single-cell transcriptomic profiling reveals B cell heterogeneity landscape

**Fig. 1:**
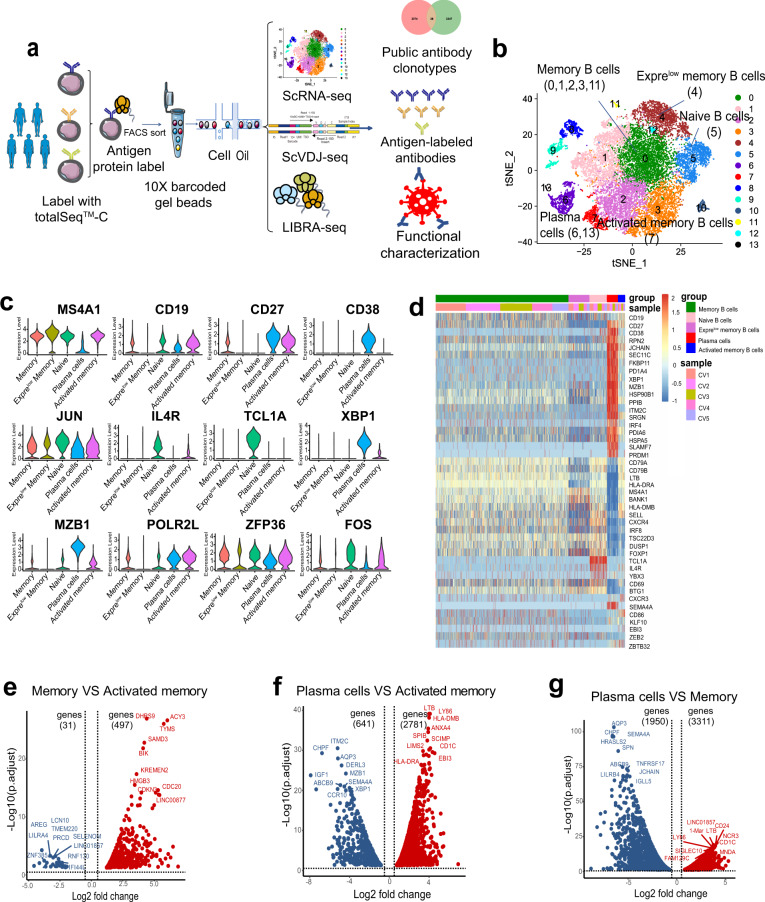
Single-Cell transcriptomic profiling reveals B cell characterization in convalescent COVID-19 patients. **a** Experiment workflow for scRNA-seq, scVDJ-seq and LIBRA-seq, including the dominant BCR clonotype, public antibody clonotypes and functional characterization. **b** Principal component analysis (PCA) and t-distributed stochastic neighbor embedding (tSNE) separates cells into 14 clusters by gene expression profile. The numbers in the brackets are the corresponding clusters for each cell type. **c** Expression levels of cell typing marker genes are shown with violin plots in five B cell subgroups. The rows represent different B cell subgroups and the columns represent the expression levels of selected marker genes. **d** Heatmap showing the cell marker genes by five B cell subgroups (memory B cells, naive B cells, Expre^low^ B cells, plasma cells and activated memory B cells) among five COVID-19 patients (CV1, CV2, CV3, CV4 and CV5). **e** Volcano plot depiction of differentially expressed genes (DEGs) between memory B cells and activated memory B cells. DEGs (*p*-adjusted < 0.05) with a | log2(fold change) | of more than 0.5 are indicated in blue(memory) and red (activated memory). **f** Volcano plot depiction of differentially expressed genes between plasma cells and activated memory B cells. DEGs (*p*-adjusted < 0.05) with a | log2(fold change) | of more than 0.5 are indicated in blue (plasma cells) and red (activated memory). **g** Volcano plot depiction of differentially expressed genes between plasma cells and memory B cells. DEGs (*p*-adjusted < 0.05) with a | log2(fold change) | of more than 0.5 are indicated in blue (plasma cells) and red (memory)

To identify B cell transcriptome landscape, we collected peripheral blood mononuclear cells (PBMCs) from five convalescent COVID-19 patients, respectively, and sorted sub-population of B cells (*CD19*^*+*^ *CD27*^*+*^ memory B cells, and *CD19*^*+*^ *CD27*^*high*^ *CD38*^*high*^ plasma cells), the sorting strategy was shown in Fig. S1. The sorted memory and plasma B cells were then subjected to 10X Chrominum and then scRNA-seq, scVDJ-seq and LIBRA-seq were performed (Fig. 1a).

Clustering analysis revealed 14 distinct cell clusters from five convalescent patients (Fig. 1b). We also separately analyzed t-distributed stochastic neighbor embedding (tSNE) clustering on the five convalescent patients (Supplementary Fig. 2) and found no significant clustering differences among the patients. B cells were distinguished by several marker genes, including *CD19, MS4A1, CD79A* and *CD79B.*^27–29^ Five B cell subgroups were further identified using other genes by scRNA-seq: (1) *IL4R* and *TCL1A* for naive B cells; (2) high expression levels of *XBP1* and *MZB1* for plasma cells;^30^ (3) *CD27* for memory B cells; (4) *CD86, CD11c (ITGAX)* and *CD95 (FAS)* for activated memory B cells; (5) *CD27*^*-*^ and *CD45*^*+*^ for Expre^low^ memory B cells, with lower gene expression (median = 696) and lower UMIs (median = 1121) compared to memory B cells (median gene expression = 1487; UMI count = 4796) (Fig. 1c, d).

To define the transcriptome characteristics of B cell subgroups, we identified differentially expressed genes (DEGs) across memory B cells, naive B cells, plasma cells and activated memory B cells. We found that *DHRS9, ACY3, TYMS* and *ACTG1* were expressed at a higher level in activated memory B cells than in memory B cells (Fig. 1e). *XBP1* and *MZB1* were expressed higher in plasma cells compared to in activated memory B cells (Fig. 1f). *XBP1* is a transcription activator in the CREB-ATF family, and is crucial for increasing protein synthesis in plasma cells.^31,32^ *MZB1* is related to B cell proliferation and viral transcription.^33^ Moreover, *CD11c* and *CD95* genes were significantly upregulated in activated memory B cells. Compared to memory B cells, *PRDM1* and *IRF4* are upregulated in plasma cells (Fig. 1g). *PRDM1* has a central role in determining and shaping the secretory arm of mature B cell differentiation and in promoting Ig synthesis,^34^ while *IRF4* regulates immunoglobulin class switch recombination and promotes the generation of plasma cells.^35^ Furthermore, *TCL1A* and *IL4R* are upregulated in naive B cells compared to other B cell subgroups (Supplementary Fig. 2b, c, d). These data revealed transcriptome landscape of B cells in convalescent COVID-19 patients and suggested high expression levels of certain genes in the activated memory B cell subgroups.

### Activated memory B cells have a higher proportion of SASRS-CoV-2 antigen-labeled cells than do memory B cells

**Fig. 2:**
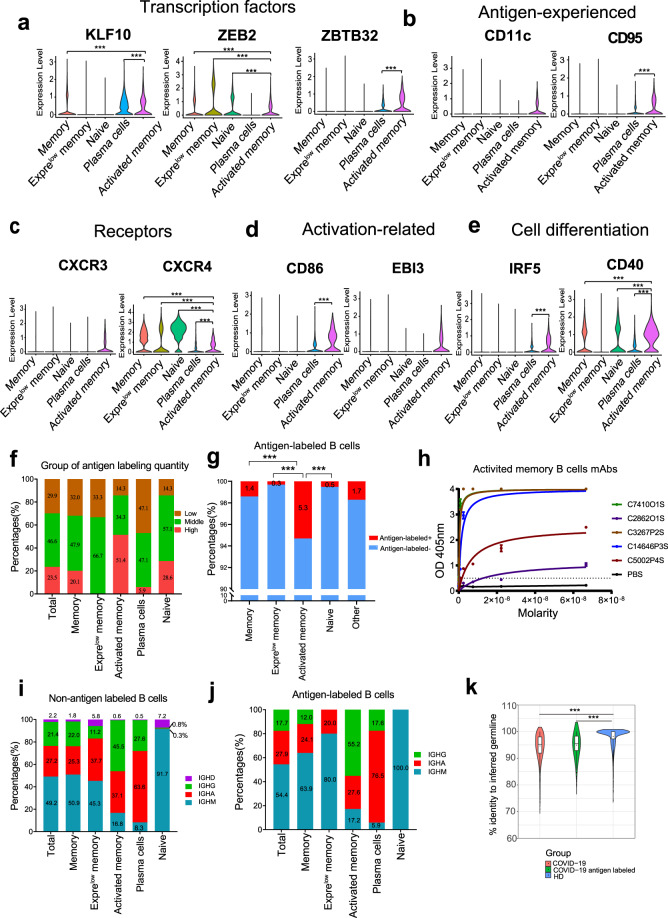
Activated memory B cells have a high proportion of antigen-labeled B cells. Gene expression distributions in distinct B cell states of established transcription factors (**a**), antigen-experienced (**b**), signaling receptors (**c**), immune activation related genes (**d**) and cell differentiation (**e**). **f** The group of antigen labeled quantity by quartile in B cell subgroups: <25% (Q_1_) showing as the low labeling quality; The 25–75% showing as the middle labeling quality; >75%(Q_3_) showing as the high labeling quality. **g** Proportion of antigen-labeled cells in memory B cells, Expre^low^ memory B cells, activated memory B cells, naive B cells and other B cells. Significance cut-offs: not significant (*p* > 0.05), *p < 0.05, **p < 0.001, ***p < 0.0001. **h** Binding of activated memory B cell antibodies to SARS-CoV-2 antigen by ELISA. PBS was used as a negative control. Data are represented as mean ± SD. Data are representative of two independent experiments. **i–j** Constituent ratio of antibody types derived from non-antigen labeled B cell subgroups (**g**) and antigen-labeled B cell subgroups (**h**). **k** Divergence from inferred germline gene sequences. The number of mutations of each clonotype relative to the inferred germline variable gene was counted. These numbers then were transformed into percent values and plotted as violin plots (COVID-19 VS COVID-19 antigen-labeled VS health)

To reveal the molecular characteristics of the B cells in convalescent COVID-19 patients, we analyzed the transcription factors, antigen-experienced, cytokine receptors, activation-related genes and cell differentiation via scRNA-seq. Transcription factors *KLF10, ZEB2* and *ZBTB32* had a higher expression level in activated memory B cells, significantly higher than those in other B cell subgroups (*p* < 0.0001, Fig. 2a). *CD11c* and *CD95*, which were reported to be associated with class switching recombination and antigen-experienced B cells,^27,36^ specifically expressed in activated memory B cells (Fig. 2b). The chemokine receptor CXCR3, which plays an important role in the migration of the cells toward the inflammation sites,^37^ is only expressed in activated memory B cells (Fig. 2c). CXCR4, controlling entry to anatomical B cell maturation location, was progressively downregulated in naive, memory, and activated memory B cells (*p* < 0.0001). Activation marker *CD86* was expressed in activated memory B cells and plasma cells (*p* < 0.0001). *EBI3*, expressed in germinal-center B cells, was found exclusively expressed in activated memory B cells in our study (Fig. 2d). Moreover, *CD40* had the highest expression level in activated memory B cells compared to other B cell subgroups (*p* < 0.0001), and *CD40* and *IRF5* were associated with human B cell activation, proliferation and plasma cell differentiation (Fig. 2e).^38,39^

Although BCR sequencing is a powerful tool for interrogating immune responses to infection and vaccination, it provides limited information about the antigen specificity of the sequenced BCRs.^22,23^ Therefore, we further performed LIBRA-seq (linking BCR to antigen specificity through sequencing) by featured barcode sequencing, which can match the naturally paired heavy and light chains with their specific antigens in a high-throughput manner. Using LIBRA-seq, a total of 235 SARS-CoV-2 antigen-labeled clones were identified in five convalescent COVID-19 patients. We pooled the labeling quantity of all antigens (RBD, S1 and NP), and took the highest labeling quantity as the labeling quantity of SARS-CoV-2 antigen, we divided them into three groups according to the quantile: low group (lower than 25%), middle group (25–75%) and high group (higher than 75%). About half of antigen labeled B cells were in the middle group, while 51.4% antigen labeled B cells belonged to the high group in activated memory B cell subgroups, compared to that 0–28.6% in other antigen labeled B cell subgroups (Fig. 2f). Furthermore, *CD11c*^*high*^ *CD95*^*high*^ activated memory B cells generated the highest percentage of SARS-CoV-2-specific BCR compared to other B cell subgroups (Fig. 2g). Antibodies from ten activated memory B cells with high level SARS-CoV-2 antigen barcode were selected and subjected to antibody production in HEK293 cells. Fifty percent (5/10) of them showed SARS-CoV-2 antigen reactivity by ELISA (Fig. 2h).

In response to SARS-CoV-2 infection, IgM, IgG and IgA were mobilized to combat the viral infection.^40^ The dominant antibody type was IgM (91.7–100%) in naive B cells (Fig. 2i, j). It is appreciated that a continuous increase of IgG proportion was reported from 19.5% at early stage of infection to 33.5% in COVID-19 at convalescent stage, as IgG antibodies were reported to have strong neutralizing ability against SARS-CoV-2 infection.^11^ In the five B cell subgroups, immunoglobulin heavy constant mu (IGHG) constituent ratio ranges from 0.8% in non-antigen labeled naive B cell subgroup to 45.5% in activated memory B cells (Fig. 2i). Antigen-labeled activated memory B cells have the highest IGHG ratio compared to other B cell subgroups (Fig. 2j). IGHA showed the highest ration in plasma cells compared to that in other B cell subgroups, especially in antigen labeled plasma cells (Fig. 2i, j). The percentage of IGHG + IGHA in activated memory B cells was higher than that in memory B cells (Supplementary Fig. 3a, b). SARS-CoV-2 RBD protein specific IgG antibodies showed the highest titer in the five convalescent patients (Supplementary Fig. 3c, d). Moreover, the number of mutations of each clone relative to the inferred germline variable gene was counted. These numbers then were transformed into percent values and plotted as violin plots. For the COVID-19 clones, values range from 71 to 100, with a median of 95, a 25th quartile of 92 and a 75th quartile of 98. For the COVID-19 antigen labeled clones, values range from 80 to 100, with a median of 95, a 25th quartile of 93 and a 75th quartile of 98. For the health, values range from 74 to 100, with a median of 100, a 25th quartile of 97 and a 75th quartile of 100 (Fig. 2k). Memory B cells from convalescent COVID-19 patients showed higher SHM rate than healthy donors (HD) (Fig. 2k). Altogether, these results indicated that *CD11c*^*high*^ *CD95*^*high*^ activated memory B cells have a higher proportion of antigen-labeled cells than other B cell subgroups.

### Non-stochastic pairing of immunoglobulin heavy and light chains variable region gene segments in convalescent COVID-19 patients

**Fig. 3:**
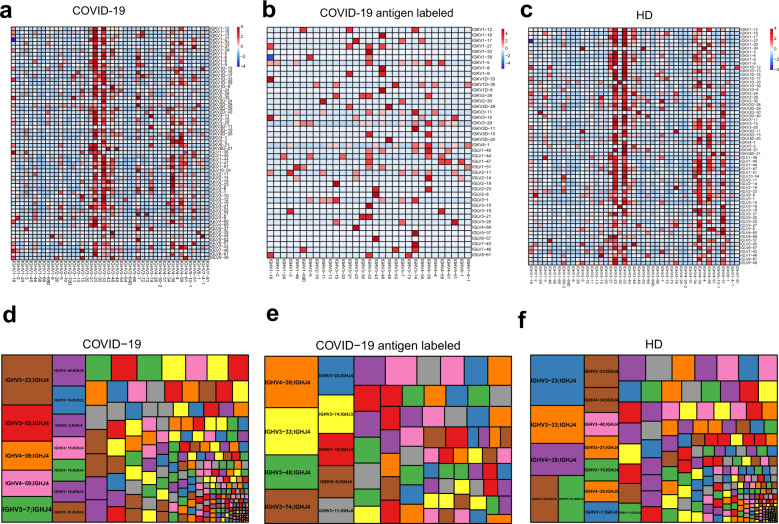
Non-stochastic paired BCR repertoire in convalescent COVID-19 patients. Heatmap showing the paired of immunoglobulin heavy chains and light chains gene variable region segments of clonotypes in convalescent COVID-19 patients. **a** COVID-19 group. **b** COVID-19 antigen labeled group (**c**) healthy donors (HD). Row and column histograms indicate marginal VH and VL frequencies, respectively. The reader color means the higher usage percentage of specific VH-VL gene pairs. Treemap showing usage frequency of specific VH-JH gene pairs in COVID-19 (**d**). COVID-19 antigen labeled (**e**) and HD (**f**). A rectangle in a treemap plot represents a unique combination of VH-JH genes. The size of a rectangle denotes the relative frequency of VH-JH gene pairs. The color of VH-JH gene pairs in each treemap plot was randomly chosen, and thus, the colors do not match between the plots

To assess the diversity of paired BCR repertoires of COVID-19 patients at the single cell level, we performed scVDJ-seq on memory B cells and plasma cells. B cells without productive IGH or IGL were filtered out. Totally, 13,964 full-length paired BCR were identified. The top 5 frequent paired heavy and light chain clonotypes are *IGHV3-74/IGKV3-11, IGHV4-39/IGKV3-20, IGHV3-23/IGKV3-20, IGHV4-59/IGKV4-1* and *IGHV4-39/IGLV2-14* (Fig. 3a). While *IGHV3-74/IGKV3-11, IGHV4-39/IGLV2-14, IGHV1-18/IGKV3-15, IGHV3-43/IGLV2-8, IGHV4-39/IGKV3-20* were the five highest frequency clonotypes in antigen labeled clones (Fig. 3b). Among them, *IGHV4-59/IGKV4-1*, antibodies (C3P2FS) showed binding activity against SARS-CoV-2 nucleocapsid protein (NP) by ELISA (Fig. 5b), consistent with previous research that *IGHV4-59/IGKV4-1* and *IGHV4-39/IGKV3-20* linkage antibodies recognized SARS-CoV-2 NP.^4^ The usage frequency of paired BCR repertoires in HD were showed in Fig. 3c. The top five clonotypes were *IGHV3-23/IGKV3-15, IGHV4-59/IGKV1D-39, IGHV3-23/IGKV1-5, IGHV3-23/IGKV1D-39, IGHV3-33/IGKV1D-39*, which were different from COVID-19 antigen labeled clones. Interesting, the top one VH-VJ gene segments of COVID-19 antigen labeled clones was *IGHV4-39/IGHJ4* (Fig. 3e). While *IGHV3-23/IGHJ4* occupied the top one both in COVID-19 total and health donors (Fig. 3d, f). Compared to IgH repertoires of healthy human donors, *IGHV3-74* and *IGLV3-1* are over-represented in heavy and light chain repertoires of antigen labeled clones respectively (Supplementary Fig. 4). The combination of V and J genes for both heavy and light chains, and the characteristics of CDRH3 sequences were shown in Supplementary Fig. 5, Fig. 6 and Fig. 7.

Our results provide an important insight into the preference of immunoglobulin heavy and light chain genes in convalescent COVID-19 patients, indicating that B cells might have undergone unique and non-stochastic V(D)J rearrangements in those patients.

### Paired immunoglobulin heavy and light chains of public antibody clonotypes shared by different COVID-19 individuals

**Fig. 4:**
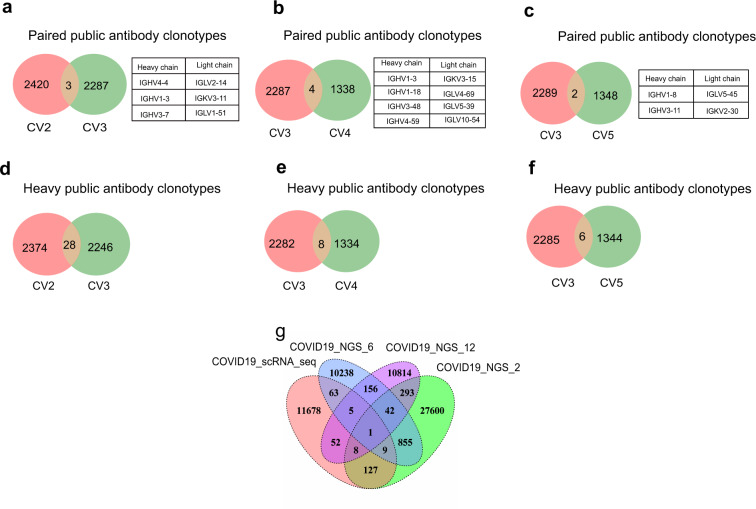
Public antibody clonotypes shared by convalescent COVID-19 patients. Public antibody clonotypes from paired immunoglobulin heavy chains and light chains shared between CV2 and CV3 (**a**), CV3 and CV4 (**b**), CV3 and CV5 (**c**), respectively. The list of particular immunoglobulin variable genes was showed on the right. Paired public antibody clonotypes were defined based on their shared VH and JH germline segments, the same VK/VL and JK/JL, and the highly similar CDRH3 amino acid sequences with 80% or greater amino acid identity. Immunoglobulin heavy chains of public antibody shared between CV2 and CV3 (**d**), CV3 and CV4 (**e**), CV3 and CV5 (**f**), respectively. The heavy chains with the same IGHV and IGHJ genes and 80% or greater similarities of CDRH3 amino acid sequences were identified as heavy chains public antibody clonotypes. **g** Public antibody clonotypes from heavy chains shared among COVID19_scRNAseq (COVID19 patients from single cell RNA sequencing), COVID19_NGS_2, COVID19_6 and COVID19_NGS_12(COVID19 patients from next-generation sequencing)

To further understanding the BCR repertoire in the context of SARS-CoV-2 infection, scRNA-seq, scVDJ-seq and next-generation sequencing (NGS) were used to learn the immunoglobulin heavy and light chains of public antibody clonotypes shared by COVID-19 individuals. Paired public antibody clonotypes were defined based on their shared VH and JH germline segments, the same VK/VL and JK/JL, and the highly similar CDRH3 amino acid sequences with 80% or greater amino acid identity.^41^ We clustered clonotypes on the samples, which shared identical IGHV-J gene and IGLV-J from all donors simultaneously (CD-HIT Methods).^42^Paired public antibody clonotypes were found among convalescent COVID-19 patients. Here we showed the paired immunoglobulin heavy chains and light chains shared between CV2 and CV3, CV3 and CV4, CV3 and CV5 (Fig. 4a, b, c). Remarkably, the amino acid sequences of some antibodies generated from different individuals were nearly identical (Supplementary table 1). For example, antibodies expressed by clonally expanded B cells with *IGHV3-11/IGKV2-30* and *IGHV1-18/IGLV4-69* recurrent in different individuals had amino acid sequence identities as high as 91% and 93%, respectively.

As heavy chain is the major region for binding to antigen, we next sought to analyze exclusively the public heavy chain in convalescent COVID-19 patients. Heavy chains with the same IGHV and IGHJ genes and 80% or greater similarities of CDRH3 amino acid sequences were identified as public heavy chains. Around 0.44–1.23% of heavy chain antibody clonotypes were shared between different COVID-19 individuals by scRNA-seq (Fig. 4d, e, f). Then, we preformed the deep sequencing of BCR heavy chain repertoires by NGS from convalescent COVID-19 patients to identify the public heavy chains in more BCR sequence sets. The percentage of shared heavy chains between scVDJ-seq and deep heavy chain sequencing ranged from 0.55 to 1.21% (Fig. 4g). Together those results highlighted public antibody clonotypes in COVID-19 patients represented an avenue of significant potential for better understanding antibody responses to SARS-CoV-2, as well as for accelerating vaccine development.

### Efficient isolation of SARS-CoV-2 reactive antibody by high-throughput LIBRA-seq

**Fig. 5:**
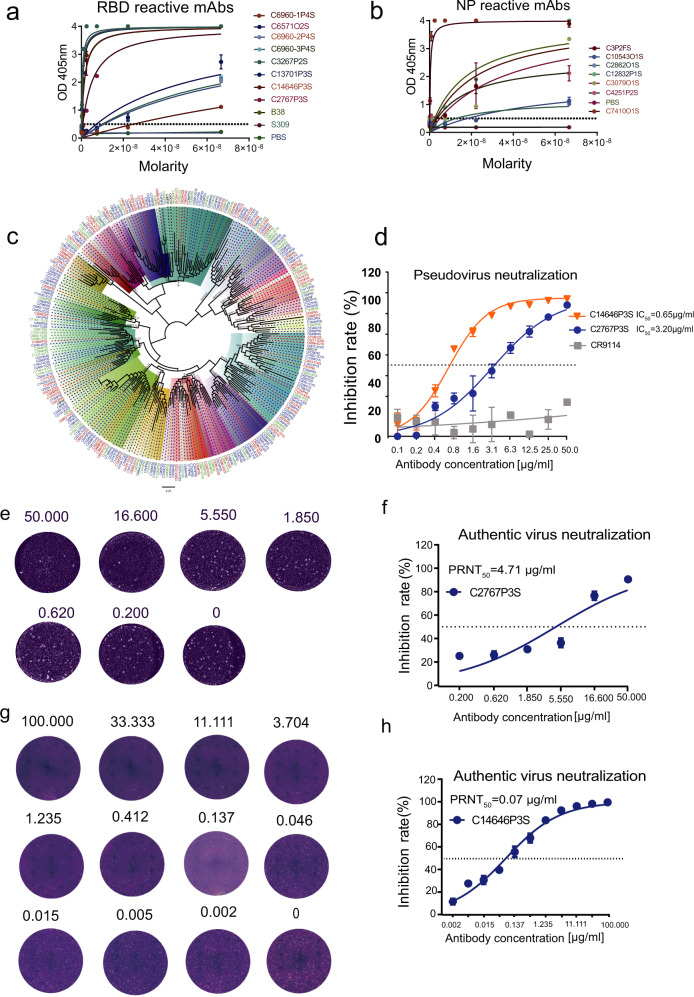
Characteristics of SARS-CoV-2 reactive mAbs. SARS-CoV-2 antigen specificity as predicted was validated by ELISA for a subset of monoclonal antibodies to SARS-CoV-2. Data are represented as mean ± SD. Data are representative of two independent experiments. **a** SARS-CoV-2 RBD reactive mAbs, (**b**) SARS-CoV-2 NP reactive mAbs. **c** Maximum-likelihood phylogenetic tree of dominant clonotypes and antigen labeled antibodies’ heavy chains. Unrooted phylogenetic tree depicting the genetic relationships among all VH genes of antigen labeled antibodies. Branch lengths were scaled so that sequence relatedness can be readily assessed. Hvdj sequences with the same VH gene usage are shown in the same color at the clades. Hvdj sequences with the same antigen-labeled quantity are shown in the same color at the branch tips (red, blue and green means high, median and low antigen-labeled quantity respectively, and black means none). **d** Neutralization of C2767P3S and C14646P3S mAbs against pseudotyped SARS-CoV-2 virus in Huh-7 cells. Influenza relative mAbs CR9114 was used as a negative control. Data are represented as mean ± SEM. Data are representative of two independent experiments. **e** C2767P3S mAb was tested using the plaque reduction neutralization assay. DMEM was used as a negative control. Data are representative of two independent experiments. **f** Neutralization of C2767P3S mAb against live SARS-CoV-2 virus in Vero E6 cells. DMEM was used as a negative control. Data are represented as mean ± SEM. Data are representative of two independent experiments. **g** C14646P3S mAb was tested using the plaque reduction neutralization assay. DMEM was used as a negative control. Data are representative of two independent experiments. **h** Neutralization of C14646P3S mAb against live SARS-CoV-2 virus in Vero E6 cells. DMEM was used as a negative control. Data are represented as mean ± SEM. Data are representative of two independent experiments

Neutralizing antibodies play a key role in guiding the vaccine development and potential therapeutics, and memory B cells are believed to be a good pool for isolating potently neutralizing antibodies. Therefore, to rapidly and effectively isolate SARS-CoV-2-neutralizing antibodies, we applied LIBRA-seq to high-throughput mapping of antigen specific B cell at the single-cell level. To validate the ability of LIBRA-seq to accurately identify SARS-CoV-2 specific B cells, 41 ideal antibody genes from 235 SARS-CoV-2 antigen-labeled B cell clones were synthetized and sub cloned into an IgG1 expression vector. Synthetic sequence sets were generated using the major rule of distinct feature cell-hashing barcode and two supplementary rules: (1) antibody isotypes except IgE and IgD; (2) high level of SHM rate is not required. Moreover, six dominant clonotypes without antigen-labeled were selected as non-ideal candidates. Via expression of the antibodies in HEK293 cells, we tested the SARS-CoV-2 reactivity of these 47 purified mAbs by ELISA. Around 56% (23/41) of mAbs with antigen labeled targeted the SARS-CoV-2 antigen (RBD, S1 or NP) (Fig. 5a, b). Among them, 54% (19/35) mAbs with high antigen labeling quantity showed the positive ELISA results, 67% (4/6) mAbs with middle antigen labeling quantity targeted the SARS-CoV-2 antigen, only 17% (1/6) clonally expanded mAbs without antigen labeled were SARS-CoV-2-specific antibodies with low ELISA binding ability. To assess the diversity and evolution signatures of VH gene usage, we constructed phylogenetic tree using the heavy chain sequences of dominant clonotypes and antigen-labeled clonotypes identified by LIBRA-seq. Unrooted phylogenetic tree depicted the genetic relationships among all of these heavy chain genes (Fig. 5c). Results revealed broad usage of VH gene families from the SARS-CoV-2-labeled clones from the five donors, which also showed a certain preference in the usage of VH genes. Among them, the VH genes with high antigen labeling quantity were mainly *VH3-53, VH3-74, VH3-30, VH3-33, VH2-5, VH1-18, VH4-39 and VH4-59*. IGHV3-53 is the most frequently used IGHV gene for targeting SARS-CoV-2 RBD in previous 294 reported mAbs.^4^ Here, more than half of *VH3-53* in our study belonged to high antigen labeling quantity (Fig. 5c).

We further characterized two RBD reactive mAbs named C14646P3S and C2767P3S from them. The mAbs exhibited neutralizing activity against SARS-CoV-2 spike pseudotyped virus, with a half maximal inhibitory concentration (IC_50_) of 0.65 µg/ml and 3.20 µg/ml, respectively (Fig. 5d). To evaluate the neutralization potential against the authentic virus, we performed the plaque reduction neutralization test (PRNT) using authentic SARS-CoV-2 isolated from COVID-19 patients (Fig. 5e, g). C14646P3S and C2767P3S mAbs neutralized authentic virus with PRNT_50_ of 0.07 µg/ml and 4.71 µg/ml respectively (Fig. 5f, h). Moreover, C14646P3S showed low levels of SHM (0.68%), with *IGHV1-69, IGKV1-12*, *IGHA1*. Compared to C14646P3S, C2767P3S showed higher SHM (1.34%), with *IGH4-39, IGLV1-40*, *IgM*. Our results showed that neutralizing mAbs against SARS-CoV-2 could be isolated via LIBRA-seq. LIBRA-seq will be an integral tool for antibody discovery and vaccine development efforts against wide range of antigen targets.

## Discussion

B cell and antibody-mediated immune response are essential to control and eliminate SARS-CoV-2 infection.^11,34,36^ Highly potent SARS-CoV-2 mAbs show several distinct signatures, including: using a broad spectrum of variable (V) genes, low levels of somatic mutations, short CDRH3 loops, and minimal affinity maturation.^4,16^ Similarly, we hypothesized that the B cells that produce the antibodies following SARS-CoV-2 infection may share some common signatures. BCR diversity offers an important approach for examining immune responses to infection.^43,44^ However, the paired BCR repertoire diversity and transcriptomic landscape of B cells after SARS-CoV-2 infection are largely unknown. Here, via scRNA-seq, scVDJ-seq and LIBRA-seq, we comprehensively characterized the B cell transcriptomic landscape, the diversity of paired BCR repertoire and the characteristic of antigen-specific B cells development at single-cell level. We found usage preference of certain immunoglobulin genes following SARS-CoV-2 infection. Several heavy chain public antibody clonotypes (*IGHV3-23/J4, IGHV3-33/J6, IGHV4-39/J4, IGHV3-7/J4, IGHV1-18/J5*) and dominant paired heavy and light chains (*IGHV4-59/IGKV4-1, IGHV3-74/IGKV3D-11*) were restrictively used by certain immunoglobulin gene segments. Importantly, two RBD-reactive antibodies C14646P3S and C2767P3S isolated by LIBRA-seq exhibited potent neutralizing activities.

Memory is the defining feature of the adaptive immune system. Immunological memory is a mechanism to protect us against reinfection. Antibodies produced by B cells are integral to this defense strategy and underlie virtually all vaccine success.^45^Studying the functionally specialized B cell subgroups has always been an interesting topic. A recent study had identified six memory B cell subgroups via mass cytometry, including *CD19*^*high*^*, CD11c*^*+*^ memory B cells and *CD95* memory B cell subgroups.^36^ *CD11c*^*+*^ B cell subgroups has been reported in the context of vaccine-elicited clonal expansion, viral infections, and autoimmunity.^27,46,47^ *CD95*^*+*^ B cell subgroups was suggested representing an effector memory population.^36^ In our study, memory B cells were classified into Expre^low^ memory B cells, memory B cells and activated memory B cells subgroups via transcriptomic analysis by scRNA-seq. Activated memory B cells were identified with both gene *CD11c*^*high*^ and *CD95*^*high*^. We found that activated memory B cells have a higher proportion of SARS-CoV-2 antigen-labeled cells than do memory B cells. Moreover, activated memory B cells contain much lower IgM portion but higher IgG portion than memory B cells subgroups, which may due to the antibody isotypes switched from IgM to IgG after virus antigen exposure. *CD95* and *EBI3* were expressed on germinal center B cells, so it suggested that activated memory B cell with *CD95* and *EBI3* expression, may emigrated recently from light zone of germinal center.^48^ Our findings indicated that activated memory B cells may play an important role in antibody response and antibody isotype switching.

BCR repertoire of B cells is diversified owing to V(D)J gene segments recombination. Hundreds of thousands of B cell V(D)J recombination provide powerful weapons against pathogen infection. Interestingly, humans share a high proportion of the same V(D)J-recombined antibody genes after contracting infectious diseases.^49^ Previous studies have reported several public antibody clonotypes in emerging virus infections.^25,50^ A recent research reported that IgA and IgG public antibody clonotypes shared between severe and mild COVID-19 individuals were identified.^40^ In this study, we identified paired heavy and light chains public antibody clonotypes shared by multiple convalescent COVID-19 patients. Public antibody clonotypes were also reported among heathy individuals.^51^ These findings suggest that some shared clonotypes may exist in different individuals, before they have been exposed to foreign antigens.^41^ While other public antibody clonotypes only appeared following exposure to foreign antigens, and may play an important role in controlling new pathogen infection and developing adaptive immune response. Targeting universal public antibody clonotypes could potentially become an efficient strategy for epitope -based SARS-CoV-2 vaccine design.

The knowledge obtained from the clonal expansion of B cell subgroups provides a better understanding of how human adaptive immune system responds to SARS-CoV-2 infection. We used LIBRA-seq to label the SARS-CoV-2 antigen-specific memory B cells and isolate SARS-CoV-2 specific mAbs. In our study, most of antigen labeled expansion clonotypes were from memory B cells and activated memory B cell subgroups. To further validate the ability of LIBRA-seq to accurately identify SARS-CoV-2 labeled B cells, we selected representative 41 BCR sequences with low-, mid- or high- level of labeling quantity of antigens. Around 56% (23/41) of the antigen labeled BCR clonotypes showed SARS-CoV-2 antigen reactivity, which may because of the non-specific binding among the barcode and B cells. Via LIBRA-seq, we had isolated two RBD-specific mAbs with neutralizing activity, which support that LIBRA-seq can be an effective method for isolating SARS-CoV-2 specific mAbs.

Overall, we comprehensively analyzed the B cell response in convalescent COVID-19 patients via scRNA-seq, scVDJ-seq and LIBRA-seq. Our analysis highlighted the usage preference toward a subset of antibody V genes and J genes after SARS-CoV-2 infection. We characterized single B cell transcriptomic landscape and identified an interesting activated memory B cell subgroup with high expression of *CD11c* and *CD95* composed of a higher proportion of SARS-CoV-2 antigen-labeled cells compared with memory B cells via LIBRA-seq. Our study provides fundamental insights into B cell response following SARS-CoV-2 infection at single-cell level and can be of value in the vaccine development against COVID-19 pandemic.

## Materials and methods

### Research subjects

All COVID-19 subjects were confirmed positive according to quantitative reverse transcription PCR (RT-qPCR). Blood samples were obtained in the Ninth People’s Hospital of Dongguan City at least 14 days after free of symptoms of COVID-19 for study. The V(D)J data of HD were downloaded from 10× genomics (https://support.10xgenomics.com/single-cell-vdj/datasets). All study procedures were approved by the Research Ethics Committee of School of Public Health (Shenzhen), Sun Yat-sen University, China. The basic information of subjects in this study showed in Supplementary table 2.

### Tissue culture

The African green monkey kidney epithelial (Vero E6) cells, the human hepatocellular carcinoma (Huh-7) cells and HEK293T cells (originally derived from female fetal cells) were maintained at 37 °C with 5% CO2 in Dulbecco’s minimal essential medium (DMEM, GIBCO) supplemented with 10% fetal bovine serum (GIBCO), 2 mM GlutaMAX and penicillin and streptomycin (100 IU/ml).

### Flow cytometry and cell sorting

Flow cytometry analysis was performed as previously described.^52^ PBMCs were isolated from blood of COVID-19 convalescent subjects using human lymphocyte separation medium. Cells were stained and mixed with fluorescent-labeled DNA-barcoded antigens and other fluorescent antibodies, and sorted by FACS with a MoFlo Astrios EQ Flow Cytometer (Beckman). The following antibodies were used to label the B cell subgroups: CD19-PB (BioLegend, Cat 302232), CD27-APC (BioLegend, Cat 302810), CD38-PE (BioLegend, Cat 303506). CD19^+^ CD27^+^ memory B cells and CD19^+^ CD27^high^ CD38^high^ plasma B cells were sorted for further scRNA-seq.

### Cell hashing

To avoid possible sample-specific batch effects, cell hashing was used to distinct samples. It uses a series of oligo labeled antibodies against CD298 and β2 microglobulin with different barcodes to uniquely label the cells from different individuals, and then they were pooled in one scRNA-seq run. Three oligo-tagged antibodies (BioLegend), GTCAACTCTTTAGCG (C0251), TGATGGCCTATTGGG (C0252), TTCCGCCTCTCTTTG (C0253) were used as cell surface label. Sorted cells from those COVID-19 convalescent subjects were pooled into one tube and stained with a mixture of oligo-labelled antibodies against target surface proteins. Briefly, ~1–2 million cells per sample were resuspended in 50 μl cell staining buffer and incubated for 10 min at 4 °C with 5 μl Fc receptor block (TruStain FcX, BioLegend) to block Fc receptor-mediated binding. Cells were subsequently incubated with mixtures of barcoded antibodies for 30 min at 4 °C, washed for three times and resuspended in PBS (0.04% BSA) for fluorescence-activated cell sorting (FACS) and then 10× library construction.

### Antigen-oligo conjugate

The SARS-CoV-2 RBD recombinant protein and nucleoprotein (NP) were expressed in HEK293T cells. The SARS-CoV-2 S1 antigen protein was purchased from Sino Biological Inc. These antigens were conjugated with oligonucleotide barcodes in-house. Briefly, SARS-CoV-2 antigens were biotinylated using the NHS-PEG4-BIOTIN (Invitrogen), according to the manufacturer’s instructions. Biotinylated antigens were then conjugated with TotalSeq-streptavidin (BioLegend, 1:100) with barcodes, 4 °C for 60 min. The following antigen barcodes were used as cell surface label: ATGCGATCAGACCGA(RBD), CAGCAACTTCATTGT(S1), CGTATGCGCTTCAAG(NP).

### 10X genomics scRNA-seq library preparation and sequencing

A total of three libraries were prepared, including scRNA-seq, scVDJ-seq and LIBRA-seq libraries. Briefly, scRNA-seq and scVDJ-seq libraries were prepared using Chromium Single Cell 5′ Library, Gel Bead and Multiplex Kit, and Chip Kit, according to the manufacturer’s instructions. Approximately 30,000 FACS-sorted B cells were washed once with 1X PBS (0.04% BSA) and the concentrations of single cell suspensions were adjusted to 800 cells/μl. After the reverse transcription (RT) step, emulsions were broken, and barcoded-cDNA was purified with Dynabeads, followed by PCR amplification (98 °C for 45 s; 13 cycles of 98 °C for 20 s, 67 °C for 30 s, 72 °C for 1 min; 72 °C for 1 min). Amplified cDNA was then used for both 5′ gene expression library construction and BCR enrichment. For gene expression library construction, 50 ng of amplified cDNA was fragmented and end-repaired, double-size selected with SPRIselect beads, and sequenced on Illumina NovaSeq 6000 platform using 150 paired-end reads. For BCR library construction, BCR was amplified with 6 cycles of PCR (98 °C for 45 s;[98 °C for 20 s, 67 °C for 30 s, 72 °C for 1 min]^2,4^ ×6; 72 °C for 1 min) followed by an additional 8 cycles of PCR (98 °C for 45 s; [98 °C for 20 s, 67 °C for 30 s, 72 °C for 1 min] x 8; 72 °C for 1 min) using DNA primers provided in the kit. VDJ region-enriched libraries were size selected with SPRI beads and sequenced on an Illumina NovaSeq 6000 instrument using 150 paired-end reads.

LIBRA-seq was prepared with the chromium single cell 5′ feature barcode library kit according to manufacturer’s instructions. Briefly, sample index PCR was performed by using an SI primer and SPRIselect reagent was used to size separate antigen barcode libraries from cellular mRNA libraries. Sample preparation for the cellular mRNA library continued according to 10× Genomics-suggested protocols, resulting in Illumina-ready libraries. We sequenced antigen barcode libraries on a NovaSeq 6000 instrument.

### Processing of scRNA-seq data

The raw fastq files were processed using the 10× Genomics Cell Ranger Software Suite (Version 3.1.0) using GRCh38-3.0.0 as reference. Feature-barcode matrices were generated using the “cellranger count” function with default parameters. Low-quality genes and cells were filtered by removing cells with (1) expressed genes fewer than 200, (2) expressed genes more than 5000, (3) mitochondrial gene percentage >10% and (4) genes expressed in <3 cells.

### Dimensionality reduction, clustering and differential gene expression analysis

The R package Seurat V3.1 was used for data scaling, transformation, clustering, dimensionality reduction, differential expression analysis.^53^ For demultiplexing based on hashtag we used the HTODemux function within the Seurat package. The filtered gene-barcode matrix was first normalized using ‘LogNormalize’ method. The top 2000 variable genes were then identified using the ‘vst’ method in Seurat FindVariableFeatures function. Principal component analysis (PCA) was performed using variable genes, and the top 18 principal components (PCs) were used to perform tSNE to visualize the cells. The resolution was set to 0.5 for clustering (with the perplexity parameter equal to 30). Cellular identity was determined by finding DEGs for each cluster using Seurat’s implementation of the Wilcoxon rank-sum test (FindMarkers), DEGs were determined with the threshold adjusted *p* value < 0.05 and absolute logged fold-change (Log2FC) ≥ 0.5. To identify cluster specific marker genes, DEGs analysis was done between each cluster to the union of the rest of the group.

### ScVDJ-seq data processing and analysis

Full-length BCR V(D)J segments were enriched from amplified cDNA from 5′ libraries via PCR amplification according to the manufacturer’s protocol (Chromium Single Cell V(D)J Reagent Kits, 10× Genomics). The BCR sequences for each single B cell were assembled by cellranger vdj pipeline (v3.1.0) for the identification of CDR3 sequence and the rearranged BCR gene with refdata-cellranger-vdj_GRCh38_alts_ensembl-3.1.0 as reference. In brief, a BCR diversity metric, containing clonotype frequency and barcode information, was obtained. Using barcode and raw clonotype id information, productive BCR clonotypes were filtered and their raw counts were inserted into the Seurat object for projecting on a t-SNE plot, R package ggplot2 were using to mapping the t-SNE plot location (*X*-axis and *Y*-axis) with BCR characters or antigen labeled clonotypes by cell barcode. Moreover, processed FASTA sequences from scVDJ-seq were annotated using IgBlast v1.16.0 against human IMGT reference database. The Hvdj sequences with antigen label obtained at that step were aligned by AliView, then processed in MegaX using a Maximum likelihood method to generate the phylogenetic trees.^54^ To identity the public antibody clonotypes, paired immunoglobulin heavy and light sequences annotated same VJ usage (not considering alleles) were clustered based on 80% CDR3 amino acid sequence similarity using CD-hit method.^42^

### BCR repertoire sequencing

RNA extraction of PBMCs was performed following RNeasy Plus Mini Kit (Qiagen). One common forward primer adaptor and one reverse primer corresponding to the constant (C) regions of each of the IGH (IgG\IgA\IgM\IgD\IgE) were designed to facilitate PCR amplification of cDNA sequences in a less biased manner. Samples were analyzed by High-throughput sequencing of BCR using the ImmuHub^®^ BCR profiling system at a deep level (ImmuQuad Biotech, Hangzhou China). Briefly, a 5′ RACE unbiased amplification protocol was used. This protocol uses unique molecular identifiers (UMIs) introduced in the course of cDNA synthesis to control bottlenecks and to eliminate PCR and sequencing errors. Sequencing was performed on an Illumina NovaSeq^®^ system with PE150 mode. One common adaptor with UMI was added on the 5′ of cDNA during the first-strand cDNA synthesize and one reverse primer corresponding to the constant (C) regions of each of the IGH were designed to facilitate PCR amplification of cDNA sequences in a less biased manner. The UMI attached to each raw sequence reads were applied to correct PCR and sequencing errors correction and PCR duplicates removing. V, D, J and C segments were mapped with IMGT^®^ and then CDR3 regions were extracted and clonotype were assembled for all clones.

### Monoclonal antibody expression and purification

Antibodies were generated as previously described.^52,55^ A total of 47 mAbs (paired heavy and light chains) were synthesized according to antigen labeling quantity and the high frequency of VH-VJ combined. The synthesized mAbs were cloned into expression vectors containing the IgG constant regions of human using SalI and XhoI restriction enzymes (Monad MF02201 and MF03001). IgG mAbs were expressed by transfecting HEK293T cells with equal amounts of heavy- and light-chain plasmids using polyethylenimine(PEI) and cultured for 5 days. The supernatant was collected and purified using protein A agarose beads.

### Enzyme linked immunosorbent assay (ELISA)

High-protein binding microtiter plates (Costar) were coated with 2 µg/ml SARS-CoV-2 RBD, S1, and NP recombinant protein in PBS overnight at 4 °C, respectively. After blocking with 3% BSA in 1 × PBS, serially diluted (1:3 ratio) mAbs starting at 10 µg/ml (or 1:100 COVID-19 convalescent plasma cells) were added into the plates and incubated for 1 h at 37 °C. Plates were washed six times with PBST and then incubated with HRP (horse radish peroxidase)-conjugated goat anti-human IgG (or IgA and IgM) (JACKON). The plate was developed with Super Aquablue ELISA substrate (eBiosciences). Absorbance was measured at 405 nm on a microplate spectrophotometer (BioTek).

### Pseudotyped virus neutralization assay

HEK293T cells were co-transfected with 60 μg of plasmid encoding Env-defective, luciferase expressing HIV-1 (pNL4-3.luc.RE) and 20 μg of SARS-Co-2-Spike into 15 cm cell culture dish. The supernatant was harvested 72 h post-transfection, centrifuged at 1000 rpm and frozen at −80 °C. To detect the neutralizing activity of antibodies, serial dilutions of mAbs were mixed with the equal volume of 650 50% tissue culture infectious doses (TCID_50_) virus of SARS-CoV-2 pseudotyped virus into a 96 well-plate and incubated for 1 h at 37 °C. Pre-prepared Huh-7 cells (100 μl, 2 × 105 in DMEM) were added to all wells and incubated for 24 h at 37 °C supplied with 5% CO2. Luciferase activity was analyzed by the luciferase assay system (Promega). The inhibition of SARS-CoV-2 pseudovirus was presented as % inhibition. IC_50_ were determined by a four-parameter logistic regression using GraphPad Prism 8.0 (GraphPad Software Inc.)

### Authentic SARS-CoV-2 PRNT

Plaque reduction neutralization test (PRNT) was performed using a clinical strain of SARS-CoV-2 (nCoV-2019BetaCoV/Wuhan/WIV04/2019).^1^ Briefly, threefold serial dilutions of 0.01 ml of mAbs (50–0.2 µg/ml for C2767P3S and 100–0.002 µg/ml for C14646P3S) were added to the same volume of 1000 TCID_50_ of SARS-CoV-2 and incubated for 1 h at 37 °C. The virus-antibody mixture was added to a monolayer of Vero E6 cells (ATCC, no. 1586) in a 24-well plate and incubated for 1 h at 37 °C. The mixture was then removed, and 0.5 mL of 0.9% (w/v) aquacide II (Millipore) in 1 × MEM (Pythonbio) supplied with 2% (v/v) FBS was added onto the infected cells. After further incubation at 37 °C for 4 days, the overlaid medium was decanted. Subsequently, the wells were fixed with 8% (v/v) formaldehyde for 12 h and then were stained with 0.5% (w/v) crystal violet to visualize the plaques. PRNT_50_ values were determined using four-parameter logistic regression analysis by using GraphPad Prism 8.0 (GraphPad Software Inc.). The experiments were performed in two repeats in a Biosafety Level 3 facility.

### Statistical analyses

All data analysis was performed using Prism software (GraphPad Version 8.0), R soft (R version 3.6.1) and Python (version 3.5). Mann–Whitney test and independent *t*-test were used to compare continuous variables. A *P* value ≤ 0.05 was considered statistically significant.

### Study approval

All study procedures were approved by the Research Ethics Committee of School of Public Health (Shenzhen), Sun Yat-sen University, China. Written informed consent was received from participants prior to inclusion in the study.

## Supplementary information

Rapid Isolation and Immune Profiling of SARS-CoV-2 Specific Memory B Cell in Convalescent COVID-19 Patients via LIBRA-seq

## Data Availability

The authors declare that the code and data used for the current study are available upon reasonable request.
