# Supplementary material for: Rapid isolation and immune profiling of SARS-CoV-2 specific memory B cell in convalescent COVID-19 patients via LIBRA-seq
Source: Signal Transduct Target Ther. 2021 May 17;6:195. doi: 10.1038/s41392-021-00610-7 (PMC8127497; doi:10.1038/s41392-021-00610-7)
Supplement: Supplementary file 1 — Rapid Isolation and Immune Profiling of SARS-CoV-2 Specific Memory B Cell in Convalescent COVID-19 Patients via LIBRA-seq [file 41392_2021_610_MOESM1_ESM.docx]

Supplementary Materials for

**Rapid isolation and immune profiling of SARS-CoV-2 specific memory B cells in convalescent COVID-19 patients via LIBRA-seq**

Bing He^1, #^, Shuning Liu^1, #^, Yuanyuan Wang^1, #^, Mengxin Xu^1^, Wei Cai^1^, Jia Liu^2^, Wendi Bai^1^, Shupei Ye^3^, Yong Ma^1^, Hengrui Hu^2^, Huicui Meng^1^, Tao Sun^4,5^, Yanling Li^1^, Huanle Luo^1^, Mang Shi^6^, Xiangjun Du^1^, Wenjing Zhao^7^, Shoudeng Chen^8^, Jingyi Yang^2^, Haipeng Zhu^9^, Yusheng Jie^10^, Yuedong Yang^11^, Deyin Guo^6^, Qiao Wang^12^, Yuwen Liu^13,14,15^, Huimin Yan^2^, Manli Wang^2^, Yao-Qing Chen^1,16^*

Correspondence to: [chenyaoqing@mail.sysu.edu.cn](mailto:chenyaoqing@mail.sysu.edu.cn)

**This PDF file includes:**

Figures. S1 to S7

Tables S1 to S2


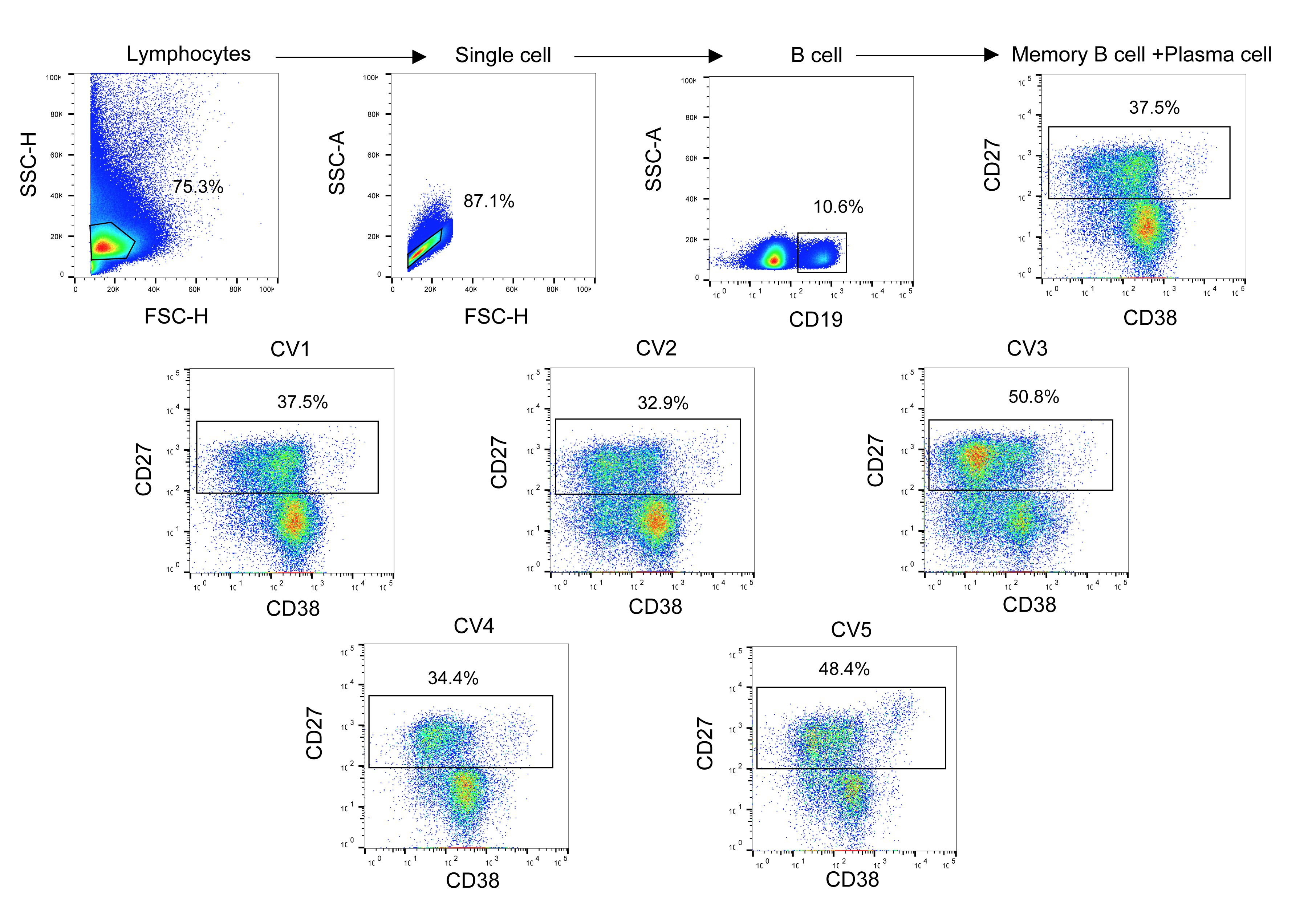


**Figure. S1 Sorting strategy on the isolation of memory B cells and plasma using flow cytometry.**


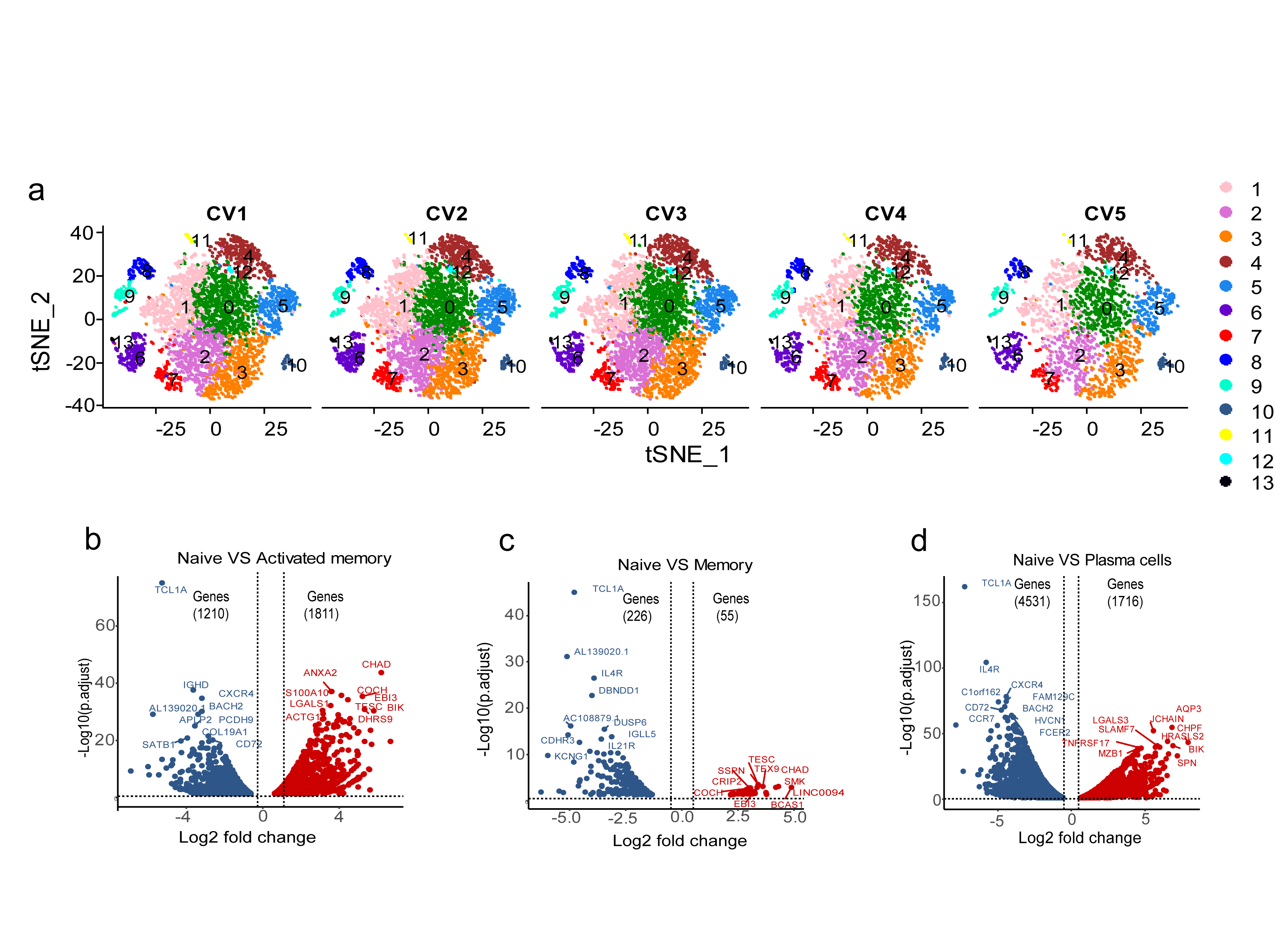


Figure S2. Characterization of gene expression in single B cells. **a** tSNE projection of 14 clusters among five convalescent COVID-19 patients. T cells (CD3E) were identified in 8 and 9 clusters, macrophage (CD68) in 10 cluster, CD19^-^ CD27^+^ cells in 12 cluster. and B cells in the rest of clusters.

Volcano plot depiction of differentially expressed genes between activated memory B cells and naïve B cells **(b)**, memory B cells and naive B cells **(c)**, and plasma and naive B cells **(d)**. DEGs (p-adjusted < 0.05) with a |log2(fold change) | of more than 0.5 are indicated in blue (up-regulated in left subgroup) and red (up-regulated in right subgroup).


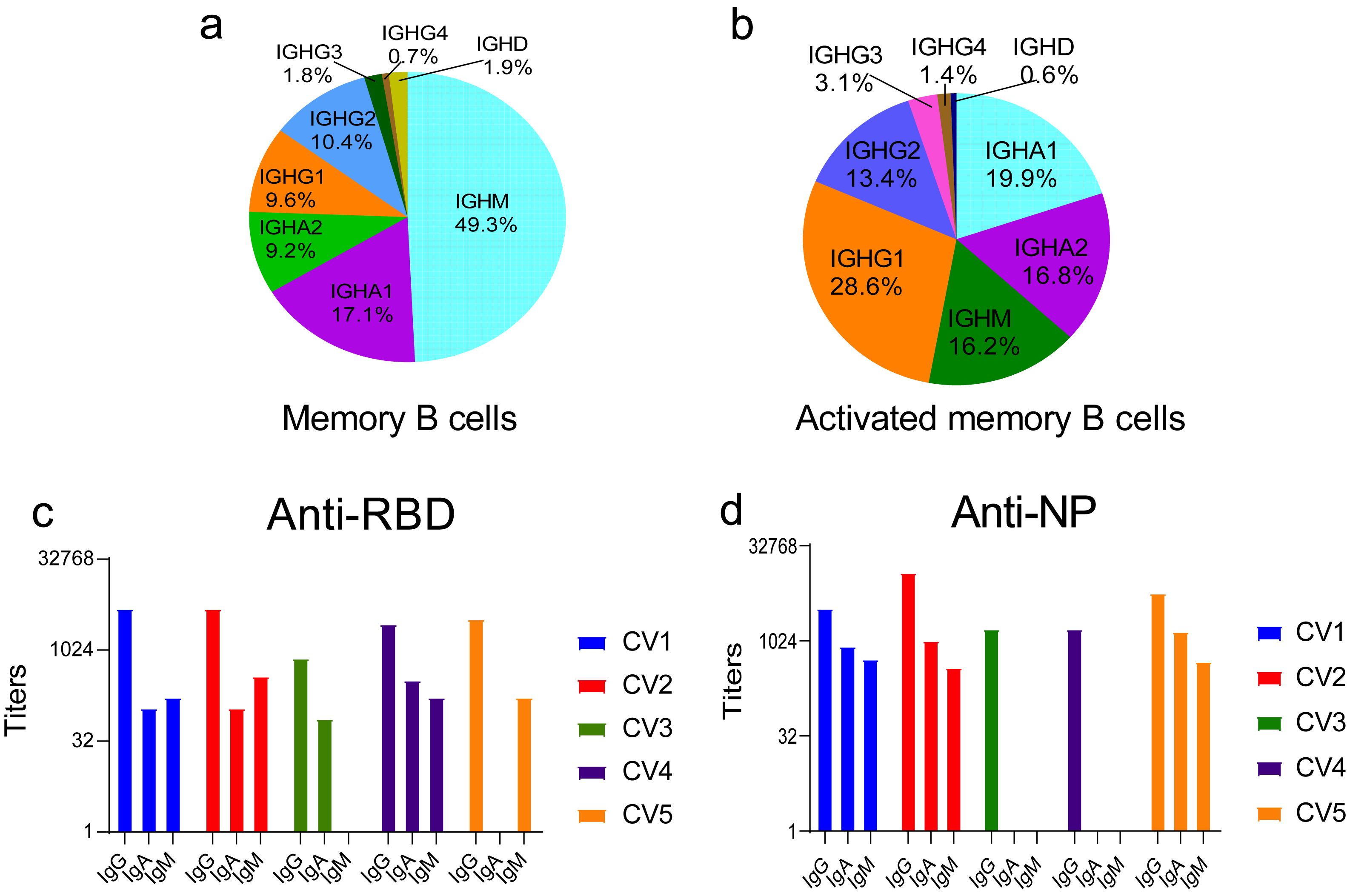


**Figure S3.** Characteristics of antibody types in five COVID-19 patients. **a** Pie chart of IgM, IgG1, IgG2, IgG3, IgG4, IgA1, IgA2 and IgD proportions in memory B cell. **b** Pie chart of IgM, IgG1, IgG2, IgG3, IgG4, IgA1, IgA2 and IgD proportions in activated memory B cell. **c** Titrations of IgG, IgA, IgM antibodies against SARS-CoV-2 RBD protein were measured by ELISA. **d** Titrations of IgG, IgA, IgM antibodies against SARS-CoV-2 N protein were measured by ELISA.


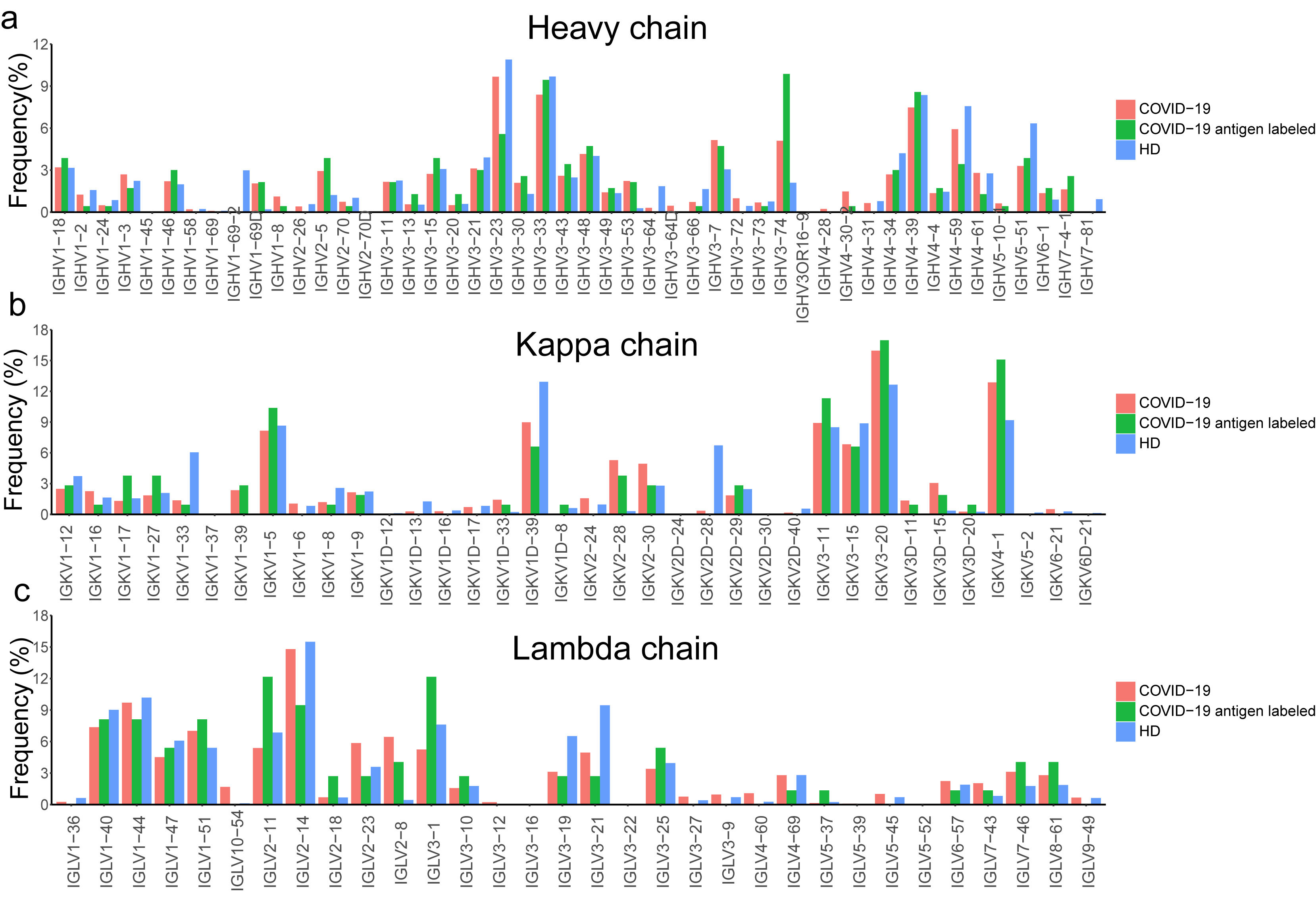


**Figure S4.** Frequency distributions of human VH, VK and VL genes. The frequency distributions of IGHV gene usage **(a)**, IGKV gene usage **(b)**, IGLV gene usage **(c)** distribution in COVID-19 antigen labeled group (green), COVID-19 total group (pink) and health group (blue).


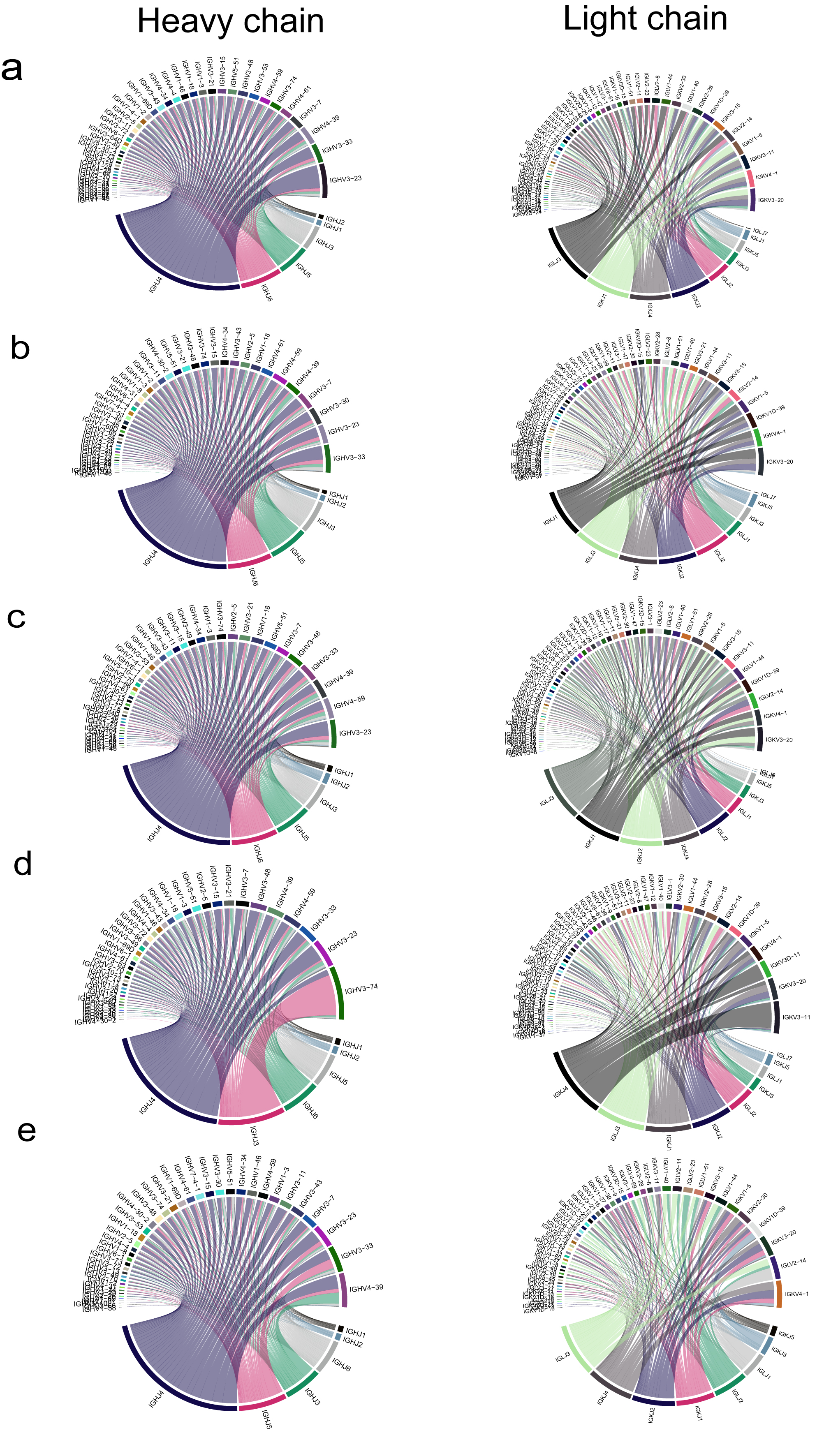


**Figure S5.** Combination of V and J gene of heavy chain and light chain.The combination of V and J gene of heavy chain and light chain in five COVID-19 patients: CV1**(a)**, CV2**(b)**, CV3**(c)**, CV4**(d)** and CV5**(e)**.


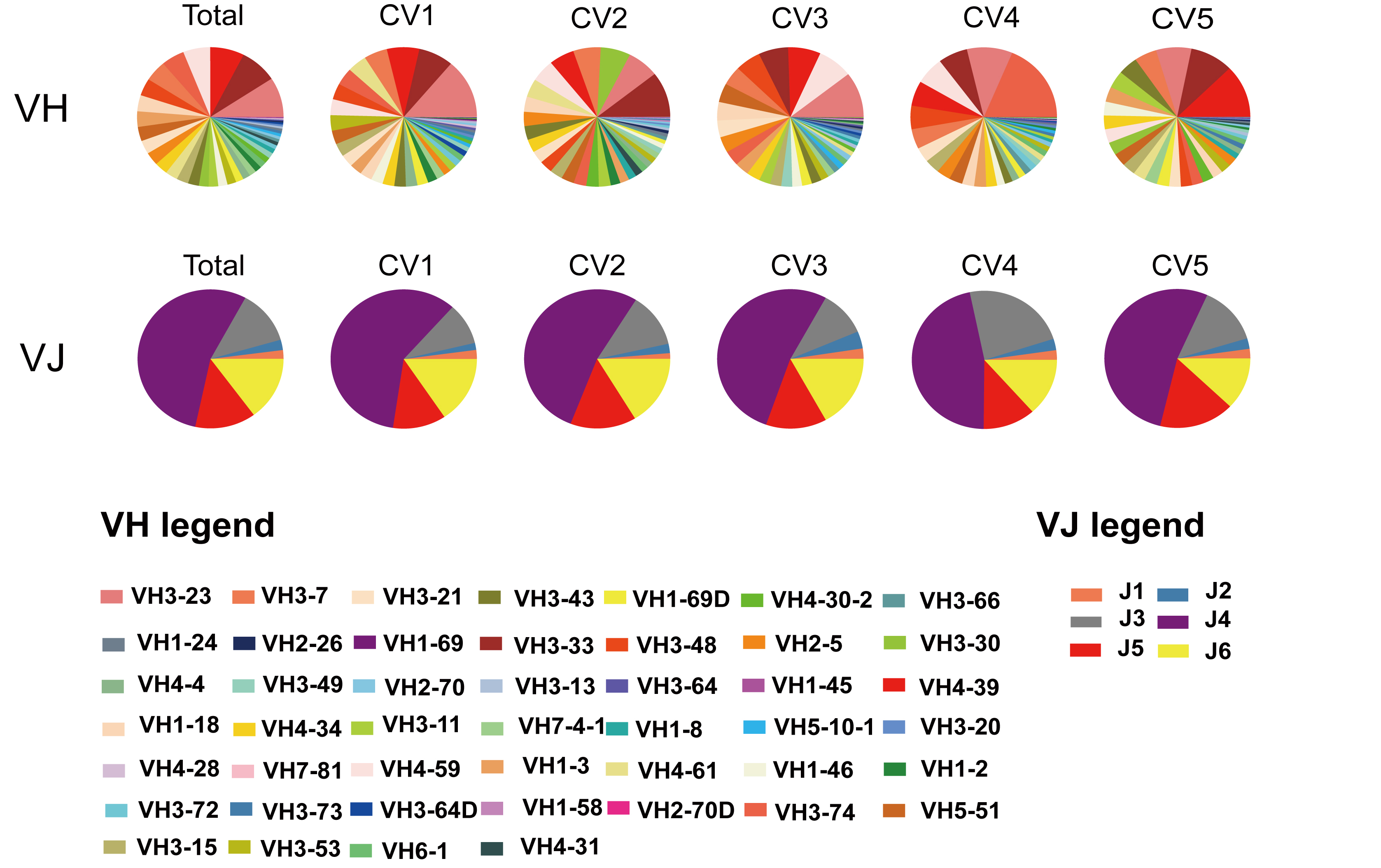


**Figure S6.** Proportion of IGHV and IGHJ genes in convalescent COVID-19 patients. Pie charts showing the combination of IGHV and IGHJ genes segments from patients CV1, CV2, CV3, CV4 and CV5, respectively.


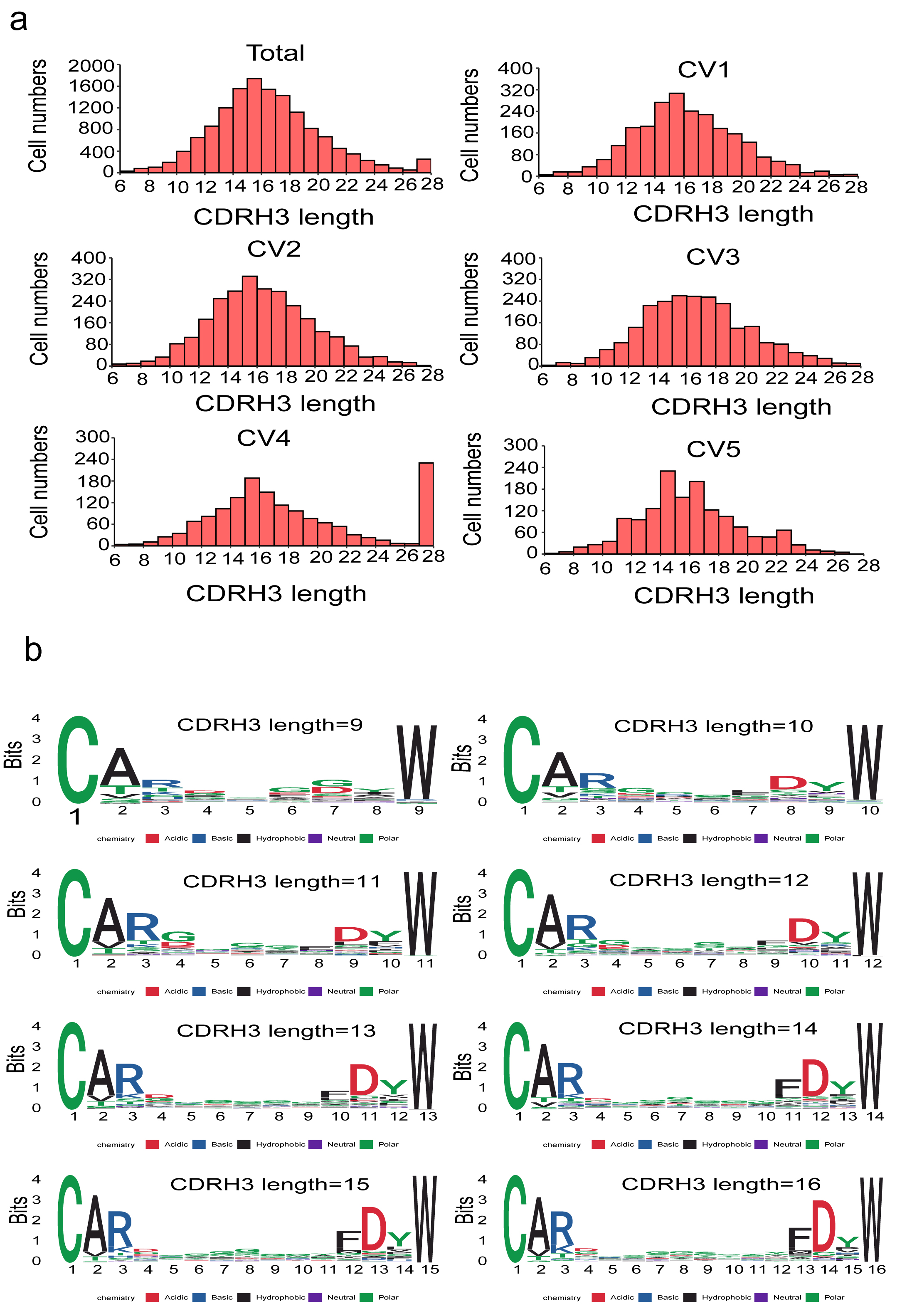


**Figure S7.** Characteristics of CDRH3 length and amino acid. **a** Histogram showing the distribution of CDRH3 length. The X-axis is the CDRH3 length, and Y-axis is the cell numbers. **b** Common CDRH3 motifs in heavy chain public antibody clonotypes with long CDRH3 (organized by CDRH3 amino acid length).

**Table S1. Information of paired public antibody clonotypes**

|  | Heavy chain | | | Light chain | | |
| --- | --- | --- | --- | --- | --- | --- |
|  | IGHV | IGHJ | CDRH3 | IGLV | IGLJ | CDRL3 |
| Paired public antibody clonotypes between CV2 and CV3 | | | | | | |
| CV2 | IGHV4-4 | IGHJ4 | CATHTYGDSTSCFDHW | IGLV2-14 | IGLJ3 | CSSYTSSSTRVF |
| CV3 | IGHV4-4 | IGHJ4 | CVTHTYGDSTSCFDSW | IGLV2-14 | IGLJ3 | CSSYTTSTTWVF |
| CV2 | IGHV1-3 | IGHJ4 | CAGGVHTDGVRW | IGKV3-11 | IGKJ4 | CQQRASWPITF |
| CV3 | IGHV1-3 | IGHJ4 | CAGGVHTDGVRW | IGKV3-11 | IGKJ4 | CQQRDNWPLTF |
| CV2 | IGHV3-7 | IGHJ4 | CARIGYSSSSTDYW | IGLV1-51 | IGLJ2 | CGTWDHSLTAVLF |
| CV3 | IGHV3-7 | IGHJ4 | CARIGYSSSSFDYW | IGLV1-51 | IGLJ2 | CGTWDGSLTAAVF |
| CV3 | IGHV3-7 | IGHJ4 | CARIGFSSSSTDYW | IGLV1-51 | IGLJ2 | CGTWDSSLTAVVF |
| Paired public antibody clonotypes between CV3 and CV4 | | | | | | |
| CV3 | IGHV1-3 | IGHJ4 | CASRPGMAAAGFDFW | IGKV3-15 | IGKJ4 | CQQYNDWPPLTF |
| CV4 | IGHV1-3 | IGHJ4 | CASRPGMAVAGLDFW | IGKV3-15 | IGKJ4 | CQQYNDWPPLTF |
| CV3 | IGHV1-18 | IGHJ5 | CARDGSGTWDDPW | IGLV4-69 | IGLJ3 | CQTWGTGIQVF |
| CV4 | IGHV1-18 | IGHJ5 | CARDGSGTWDDSW | IGLV4-69 | IGLJ3 | CQTWGTGIQVF |
| CV3 | IGHV3-48 | IGHJ4 | CAREAGQLDYW | IGLV5-39 | IGLJ3 | CAIWYSTTWVF |
| CV4 | IGHV3-48 | IGHJ4 | CAREAGPLDYW | IGLV5-39 | IGLJ3 | CAIWYSNTWVF |
| CV3 | IGHV4-59 | IGHJ4 | CARGPPPDFDYW | IGLV10-54 | IGLJ3 | CSAWDSSLYAWVF |
| CV4 | IGHV4-59 | IGHJ4 | CARGPPPAHDYW | IGLV10-54 | IGLJ3 | CSAWDFSLSAWVF |
| Paired public antibody clonotypes between CV3 and CV5 | | | | | | |
| CV3 | IGHV1-8 | IGHJ4 | CARSPPNWGFDYW | IGLV5-45 | IGKJ3 | CMIWHSSAWVF |
| CV5 | IGHV1-8 | IGHJ4 | CARGPPNWGFDYW | IGLV5-45 | IGKJ3 | CMVWHNSAWVF |
| CV3 | IGHV3-11 | IGHJ4 | CTRDPRRADFW | IGKV2-30 | IGLJ3 | CMQGTHWEFTF |
| CV5 | IGHV3-11 | IGHJ4 | CTRDPRRVDFW | IGKV2-30 | IGLJ3 | CMQGTHWPFTF |

Sample sequence alignment for antibodies originating from different individuals that display highly similar V(D)J sequences including CDR3s

**Table S2. Information of COVID-19 in the study**

| Sequence method | Subject | Sex | Age (years) | symptom | Date of symptom onset | Discharged | Date of PBMC collection |
| --- | --- | --- | --- | --- | --- | --- | --- |
| ScRNA-seq, VDJ-seq and LIBRA-seq | CV1 | Male | 29 | mild | January 27, 2020 | February 20, 2020 | February 28, 2020 |
|  | CV2 | Female | 35 | mild | January 31, 2020 | February 23, 2020 | Marth 7, 2020 |
|  | CV3 | Male | 56 | mild | February 3, 2020 | February 25, 2020 | February 25, 2020 |
|  | CV4 | Female | 59 | mild | January 16, 2020 | February 3, 2020 | February 25, 2020 |
|  | CV5 | Female | 47 | severe | January 24, 2020 | February 22, 2020 | Marth 7, 2020 |
| NGS | NGS_2 | Male | 69 | —— | January 17,2020 | April 8, 2020 | March 31, 2020 |
|  | NGS_6 | Male | 59 | —— | January 16, 2020 | March 31, 2020 | April 4, 2020 |
|  | NGS_12 | Female | 59 | —— | February 8, 2020 | April 1, 2020 | March 29, 2020 |
